# The Gender Euphoria Scale (GES): a protocol for developing and validating a tool to measure gender euphoria in transgender and gender diverse individuals

**DOI:** 10.3389/fpsyg.2023.1284991

**Published:** 2024-01-05

**Authors:** C. A. Blacklock, M. A. Tollit, C. C. Pace, B. Elphinstone, K. C. Pang, S. Buzwell

**Affiliations:** ^1^Department of Psychological Sciences, Swinburne University of Technology, Melbourne, VIC, Australia; ^2^Murdoch Children’s Research Institute, Melbourne, VIC, Australia; ^3^The Royal Children’s Hospital, Melbourne, VIC, Australia; ^4^Department of Paediatrics, The University of Melbourne, Melbourne, VIC, Australia

**Keywords:** transgender, gender diverse, gender euphoria, gender dysphoria, mental health, scale development, protocol

## Abstract

**Background:**

Gender euphoria (i.e., a positive feeling associated with one’s gender identity, expression, or affirmation) is widely discussed among transgender and gender diverse (hereafter referred to as trans) individuals. However, as a construct, gender euphoria has never been formally measured and has rarely been empirically studied. Hence, this protocol paper illustrates our process for developing and validating a new tool to measure gender euphoria, known as the Gender Euphoria Scale (GES), for use with trans populations.

**Methods:**

Deductive methods including findings from previous research and a review of existing measures, together with inductive methods such as expert feedback and focus groups with trans individuals, were used to generate a preliminary item pool for the GES. Pilot testing with trans community members and mental health clinicians was then used to refine items and develop a preliminary scale. Trans participants involved in an ongoing longitudinal study (TRANSform) were invited to complete the scale alongside measures of personality and gender factors to assess validity. Participants were then invited to complete the scale two weeks after initial completion to assess the test–retest reliability of the scale. The next stage in the scale development process will be to examine the dimensionality of the GES using exploratory factor analytic techniques. The scale will then be assessed for internal consistency, temporal stability, discriminant validity, and convergent validity.

**Conclusion:**

This paper outlines the development and characterization of a novel tool to measure gender euphoria for the first time. The GES will facilitate research opportunities to better understand the nature of gender euphoria and its influences, and may be used clinically to examine relationships between gender euphoria and gender affirming interventions. Hence, we expect the GES to make a significant contribution to both research and clinical practice with trans communities.

## Introduction

Research examining the mental health of transgender and gender diverse (hereafter referred to as trans) individuals has traditionally focused on gender dysphoria (i.e., gender-related distress) and mental health difficulties (Bradford et al., 2021). Consequently, there is a dearth of research focused on positive aspects of gender diversity. While examining mental health outcomes remains important for developing risk mitigation strategies, this illness focus has inadvertently pathologized trans individuals and their experiences (Bradford et al., 2021). To counter this, there is a need to explore and develop a comprehensive understanding of positive dimensions of mental health and gender-related experiences reported by trans individuals.

One positive experience commonly reported in trans communities, but largely omitted from the academic literature to date, is gender euphoria (Austin et al., 2022). Gender euphoria has been described by trans people as the “experience of comfort, connection and celebration related to our internal sense of self and our gender. The pride of feeling and being affirmed as who we are”.^1^ Though the construct has received little empirical attention, researchers concur that gender euphoria encompasses joyful feelings related to gender congruence and authenticity (Ashley and Ells, 2018; Beischel et al., 2022). Gender euphoria may arise in relation to a person’s gender identity, gender expression, or gender affirmation (Benestad, 2010; Ashley and Ells, 2018; Bradford et al., 2021; Beischel et al., 2022) and range in intensity between feelings of contentment to impressions of intense happiness or elation (Beischel et al., 2022; Jacobsen and Devor, 2022).

While it has been suggested that gender euphoria could be the antithesis of gender dysphoria (Ashley and Ells, 2018), recent studies indicate that gender euphoria is likely a construct in its own right. For instance, Bradford et al. (2021) found that undergoing hair removal as a gender affirming intervention provided relief from gender dysphoria and separately promoted positive affect (i.e., gender euphoria) among transfeminine adults. Additionally, two qualitative studies found that although participants reported a relationship between gender euphoria and gender dysphoria, many indicated they were distinct rather than opposing experiences (Beischel et al., 2022; Jacobsen and Devor, 2022). Such findings point to the need to examine gender euphoria and gender dysphoria as separate, albeit presumably related, constructs.

Though researchers are beginning to highlight the significance of gender euphoria (Beischel et al., 2022), it has yet to be adequately operationalized and measured as a construct. Developing a tool to measure gender euphoria could facilitate opportunities to reframe research to examine positive outcomes for trans individuals, rather than focusing solely on mitigating poor outcomes as has often been the case. Furthermore, from a clinical perspective, gender euphoria has been found to be a driver for some trans individuals to seek gender affirming medical interventions, including hormonal treatments and surgery (Ashley and Ells, 2018; Beischel et al., 2022). Having a tool to assess gender euphoria in the context of gender affirming medical interventions would therefore enable a holistic assessment of these procedures and their ability to promote wellbeing among trans individuals. Finally, identifying social factors that enhance or inhibit gender euphoria, could highlight avenues for interventions to improve mental health. Taken together, there is an urgent need to develop a tool to measure gender euphoria.

With these broader objectives in mind, we will develop and validate a tool to measure gender euphoria for use with trans individuals. In doing so, we aim to create a tool which measures the frequency, intensity, and stability of gender euphoria, in addition to identifying experiences that contribute to gender euphoria. Moreover, we intend for this tool to be sensitive to change, relevant to people of all trans gender identities, and suitable for use in both research and clinical settings.

## Methods and analysis

Our process for developing a scale to measure gender euphoria is based on best practice guidelines (Boateng et al., 2018) and well-established methods for developing scales within the social sciences (DeVellis, 2021). The three stages outlined below include: (1) item development, (2) scale development, and (3) scale evaluation.

### Item development

#### Domain identification

To facilitate item generation, the domain of interest must first be defined (Haynes et al., 1995). Though relatively little research has examined gender euphoria, Citron et al. (2020) developed a definition of gender euphoria based on a systematic review of existing literature and qualitative research with 395 trans adults. According to Citron et al. (2020), gender euphoria encompasses three main aspects: (1) positive affect, (2) feelings of gender belonging, and (3) feeling authentic to oneself. In their follow up study, the authors also identified a number of themes relevant to experiencing gender euphoria including congruence, gender expression, medical affirmation, positive emotions, being affirmed by others, and self-affirmation (Citron et al., 2020). These themes were used to guide item generation for our preliminary scale.

#### Item generation

The aim in generating an initial item pool is to increase the chances of tapping all dimensions of a construct (DeVellis, 2021). To this end, both deductive and inductive methods were used to generate items, to capture as many potential facets of gender euphoria as possible. In addition to developing items based on themes from previous research (Citron et al., 2020), we undertook a review of existing tools measuring psychological constructs among trans adults. To do this, we conducted a keyword search in APA Psych Tests and cross referenced the results with a systematic review of measures developed for trans adult populations (Shulman et al., 2017). The results of our review confirmed that no existing tools explicitly measured gender euphoria. Nonetheless, a number of items related to gender euphoria were identified, such as items measuring positive emotions, gender affirmation, and gender congruence. These items were subsequently added to the item pool.

#### Content validity

Content validity evaluates the extent to which a tool measures the construct of interest (Koller et al., 2017). To assess the content validity of the initial item pool, experts in the field of trans mental and physical health (*n* = 4) and trans researchers with lived experience of gender euphoria (*n* = 4) were consulted. During this process, items were reviewed and refined, resulting in a preliminary scale comprising 65 items related to frequency, intensity, and stability of gender euphoria in addition to questions relating to experiences leading to gender euphoria.

### Scale development

#### Preliminary scale piloting

Piloting a scale allows for identification of potential problems that need to be modified prior to the scale being administered to a larger sample (Dawis, 1987). To ensure the preliminary scale accurately reflected the experiences of trans individuals, was clear and comprehensible, it was administered to a trans community advisory group (*n* = 8) established through the TRANSform study.^2^ The TRANSform study is an ongoing longitudinal study investigating the health and wellbeing of trans Australians across the lifespan, and their community advisory group comprises members from underrepresented trans communities including First Nations people, people who are neurodivergent, and people with lived experience of disability. TRANSform advisory group members were invited to provide feedback on the items in addition to answering specific questions regarding the clarity of the instructions, relevance of the items, response options, administration time, and the emotional experience of completing the scale. Feedback from the advisory group was used to refine the definition of gender euphoria and response format used for the scale. In addition, items were included to capture experiences of gender euphoria related to intimacy, meeting others with similar gender identities, and seeing trans people represented in the community, media, and workplace. Clinicians working in trans mental and physical healthcare (*n* = 8) were then emailed the revised scale and asked to provide feedback. This resulted in a survey comprising 16 questions related to frequency, intensity, and stability of gender euphoria and a 66-item scale comprising questions relating to experiences contributing to gender euphoria.

#### Survey administration

Between November 21st and December 23rd 2022, the preliminary GES was administered to the TRANSform cohort. Participants in TRANSform study were recruited through various avenues including LGBTQIA+ community organizations, gender clinics, and social media (e.g., Facebook). Participants are eligible to participate in the study if they are aged 16 years or older, reside in Australia, and have a gender other than what was presumed for them at birth or a cultural gender identity different to man or woman. TRANSform participants are invited by email to participate in annual surveys. The preliminary GES and validation measures were administered as one of these surveys. Participation was voluntary, and participants were informed that they could opt out at any time prior to survey submission. Participants were also notified that survey completion implied consent to participate, with responses contributing to research. In case of potential distress arising from the survey, participants were presented with a list of supports, including LGBTQIA+ specific groups and helplines, prior to and after completing the survey.

Recruitment via the TRANSform cohort, resulted in a total sample of 732 participants, meeting the recommended 10 participants per item for exploratory factor analysis (Yong and Pearce, 2013). In addition to completing the preliminary GES, participants in the TRANSform cohort were invited to complete two measures to assess the discriminant and convergent validity of the GES – the Mini International Personality Item Pool (Donnellan et al., 2006) and the Transgender Congruence Scale (Kozee et al., 2012). Use of the latter two scales to assess validity is described below. Following administration of the preliminary GES and validity measures, participants were asked for their consent to be contacted two weeks later to complete the preliminary GES a second time. This timeframe, based on recommendations outlined by Streiner et al. (2014), will allow for eventual assessment of the test–retest reliability of the scale.

#### Data collection and management

The preliminary GES, Mini International Personality Item Pool, and Transgender Congruence Scale were administered via the web-based survey platform REDCap (Harris, 2009). Participants currently enrolled in the TRANSform study were emailed a link with an invitation to complete an online survey comprising the preliminary GES and the two validity measures. The GES was then re-administered two weeks later via REDCap (Harris, 2009) to those who consented to be recontacted. This data is now stored securely on a password-protected server that can only be accessed by the study investigators. The data will be stored for 7 years following project completion and any data that is published will be non-identifiable group data, thus ensuring the anonymity of participants.

#### Item reduction and factor extraction

With data now collected, an exploratory factor analysis will next be performed to identify the items that most contribute to the measurement of gender euphoria. Identifying the factor structure of the scale will in turn allow for potential subscales to be revealed. During this process, problematic items, such as those with low shared variance, will be eliminated, as such items do not adequately discriminate between respondents with varying levels of the construct (i.e., gender euphoria) and are therefore unlikely to contribute to the overall scale (DeVellis, 2021). Given recommendations that the initial item pool be two to five times greater than that of the final scale (Weiner et al., 2012; Kline, 2013), it is thus likely that our final number of items will be reduced to 20–40, which should be comprehensive enough to capture the potentially multifaceted nature of gender euphoria, while also being brief enough to minimize respondent burden and maximize response rates (Morgado et al., 2017).

Assuming that items are not severely skewed, the maximum likelihood method will then be used to extract factors. Oblique rotation will also be used, as items are expected to be correlated. To avoid overestimation of factors retained, factors will be determined by examining the Scree Plot (Cattell, 1966) in conjunction with Kaiser’s Criterion (Kaiser, 1960) which recommends retaining factors with eigenvalues greater than one.

### Scale evaluation

#### Reliability

To assess the reliability of the GES, both the internal consistency and temporal stability of the scale will be examined. The Greatest Lower Bound will be used to assess internal consistency as other methods (e.g., McDonald’s omega) have been found to overestimate reliability in large samples (Malkewitz et al., 2023). To assess temporal stability, Pearson’s *r* will be used to examine correlations between scores across separate administrations of the GES. A correlation between 0.70 and 0.80 will indicate adequate test–retest reliability (Taylor, 1990).

#### Validity

The final step in the scale evaluation process will be to assess the discriminant and convergent validity of the GES. This will involve examining correlations between the GES and theoretically unrelated and related measures. As gender euphoria has not been empirically linked to personality, discriminant validity will be assessed using the Mini International Personality Item Pool. The Mini International Personality Item Pool is a 20-item measure of the ‘Big Five’ personality traits (Goldberg, 1993). Responses, rated on a 5-point scale, range from 1 = very inaccurate to 5 = very accurate. Scores for each of the five personality traits are summed, with higher scores reflecting stronger identification with the respective trait. As extraversion, conscientiousness, openness, agreeableness, and neuroticism have been linked to constructs potentially related to gender euphoria, such as subjective wellbeing (DeNeve and Cooper, 1998; González Gutiérrez et al., 2005), social wellbeing (Hill et al., 2012), and self-esteem (Amirazodi and Amirazodi, 2011) we may expect to see theoretically meaningful correlations between the GES and these personality traits. However, non-significant correlations between the GES and each of the five personality subscales will indicate that gender euphoria is conceptually distinct from these personality traits.

Additionally, as previous research indicates that gender congruence, or a feeling of “rightness,” is related to gender euphoria (Ashley and Ells, 2018; Beischel et al., 2022) the Transgender Congruence Scale will be used to assess the convergent validity of the GES. The Transgender Congruence Scale is a 12-item measure of the degree to which a person accepts and experiences congruence between their physical appearance and gender identity. Responses, rated on a 5-point scale, range from 1 = strongly disagree to 5 = strongly agree. Scores are summed, with higher scores indicating higher levels of gender identity acceptance and congruence. Hence, a strong positive correlation between the GES and the Transgender Congruence Scale will be indicative of convergent validity. Convergent and discriminant validity will be assessed using Pearson’s *r* (Norman, 2010). The construct validity of the GES will be established if it correlates as predicted with the aforementioned measures.

#### Community involvement

Trans community involvement has played an integral role in the development of the GES. The decision to create a tool to measure gender euphoria was based on calls from trans community members to have ways of formally evaluating trans-specific positive life experiences (LeBlanc et al., 2022). Previous qualitative research with trans people was then used to identify key themes related to their experiences of gender euphoria. Once a preliminary item pool was established, this was disseminated to trans researchers who assisted in refining the item pool and developing a preliminary scale. Focus groups with trans people from underrepresented communities were then used to refine the items and response options for the scale. Participants in these focus groups were reimbursed for their time.

Once the preliminary scale was developed, it was then administered to the TRANSform cohort. The TRANSform study is run by researchers who identify as gender diverse and who continuously seek feedback from their trans participants regarding research priorities, ethical data collection, and dissemination of findings. All studies administered to the TRANSform cohort must contribute to improving the health or well-being of the trans community, and we believe the current study aligns with this objective.

## Discussion

Currently, there are only a few tools to measure positive experiences within the trans community (e.g., the Transgender Positive Identity Measure; Riggle and Mohr, 2015). Though critical to examine negative life experiences and how to mitigate them, the lack of tools for evaluating positive aspects of gender diversity inadvertently contributes to a deficit-based narrative of trans existence. To counter this negative, unidimensional depiction, trans people have requested that researchers expand narratives of transness to incorporate elements of positivity and joy (LeBlanc et al., 2022; Corbett, 2023).

Gender euphoria is a positive experience that has been identified as an important construct to trans people (Austin et al., 2022). However, to date, gender euphoria has never been formally measured. To remedy this gap, the current paper describes a protocol for developing and validating a tool to measure gender euphoria. The GES will be designed to measure multiple aspects of gender euphoria including frequency and intensity of gender euphoria, as well as the experiences that promote gender euphoria. The scale will also be sensitive to changes in levels of gender euphoria over time and relevant to trans people with binary and nonbinary gender identities.

In addition to developing a novel tool that measures a positive dimension of gender diversity, a major strength of this project is its commitment to trans community participation and inclusion. Collaboration with trans community members, including those from typically underrepresented trans communities, will help to ensure that the GES accurately reflects the needs and experiences of the target population. A potential limitation of this tool, however, is that the GES will be validated on a sample of trans individuals aged 16 years and older. Thus, future research should consider adapting the scale for use with younger populations. Further validation studies may involve examining the relationship between the GES and other tools measuring positive aspects of gender diversity such as the Transgender Positive Identity Measure (Riggle and Mohr, 2015). This could allow for a comprehensive investigation into the convergent validity of the GES, especially given it is unclear to what extent responses to the Transgender Congruence Scale assess gender euphoria.

Our aim in developing the GES is to provide research opportunities to better understand the nature of gender euphoria and the factors impacting its occurrence. The GES may also be used in clinical settings to examine relationships between gender euphoria and gender affirming medical care. Having such a scale may in turn facilitate a more holistic approach to clinical care with trans people and hopefully minimize the focus on proving distress in order to access gender affirming medical care. Accordingly, the GES is expected to make a significant contribution to both research and clinical practice with trans communities.

### Ethics and dissemination

All procedures conducted and proposed are consistent with the ethical standards of the Institutional Review Bodies of the Austin Health Human Research Ethics Committee (HREC/57155/Austin-2019), Thorne Harbor Health Community Research Endorsement Panel (THH/CREP 20-006D), and the ACON Research Ethics Review Committee (RERC 2020/03) and have been approved accordingly. Once validated, the GES will be made available to all participants involved in the current study. The GES will also be disseminated to trans community members, clinicians, and researchers via peer reviewed journals, conferences, and by request.

## Ethics statement

The studies involving humans were approved by all procedures conducted and proposed are consistent with the ethical standards of the Institutional Review Bodies of the Austin Health Human Research Ethics Committee (HREC/57155/Austin-2019), Thorne Harbor Health Community Research Endorsement Panel (THH/CREP 20-006D), and the ACON Research Ethics Review Committee (RERC 2020/03) and have been approved accordingly. The studies were conducted in accordance with the local legislation and institutional requirements. The participants provided their written informed consent to participate in this study.

## Author contributions

CB: Conceptualization, Investigation, Methodology, Writing – original draft. MT: Conceptualization, Methodology, Supervision, Writing – review & editing. CP: Conceptualization, Methodology, Supervision, Writing – review & editing. BE: Methodology, Supervision, Writing – review & editing. KP: Conceptualization, Funding acquisition, Methodology, Supervision, Writing – review & editing. SB: Conceptualization, Methodology, Supervision, Writing – review & editing.

## Footnotes

^1^Transhub Language (2021). TransHub. https://www.transhub.org.au/language. [Accessed August 14, 2023].

^2^Ongoing Research Transform (no date). Trans Health Research. Available at: http://www.transresearch.org.au [Accessed: August 14, 2023].
